# Identification and expression of differentially expressed genes in the hard clam, *Mercenaria mercenaria*, in response to quahog parasite unknown (QPX)

**DOI:** 10.1186/1471-2164-10-377

**Published:** 2009-08-14

**Authors:** Mickael Perrigault, Arnaud Tanguy, Bassem Allam

**Affiliations:** 1 School of Marine and Atmospheric Sciences, Stony Brook University, Stony Brook. NY 11794-5000, USA; 2 UPMC Université Paris 6, UMR 7144, Equipe Génétique et Adaptation en Milieu Extrême, Station Biologique de Roscoff, 29682 Roscoff, France and UPMC Université Paris 6, UMR 7138, Systématique, Adaptation et Evolution, 75005 Paris, France

## Abstract

**Background:**

The hard clam, *Mercenaria mercenaria*, has been affected by severe mortality episodes associated with the protistan parasite QPX (Quahog Parasite Unknown) for several years. Despite the commercial importance of hard clams in the United States, molecular bases of defense mechanisms in *M. mercenaria*, especially during QPX infection, remain unknown.

**Results:**

Our study used suppression subtractive hybridization (SSH), as well as the construction of cDNA libraries from hemocytes to identify genes related to the defense of the hard clam against its parasite. Hard clams were experimentally infected with QPX and SSH was performed on mRNA samples extracted from mantle and gill tissues at different times post-challenge. A total of 298 clones from SSH libraries and 1352 clones from cDNA libraries were sequenced. Among these sequences, homologies with genes involved in different physiological processes related to signal transduction, stress response, immunity and protein synthesis were identified. Quantitative PCR revealed significant changes in the expression of several of these genes in response to QPX challenge and demonstrated significant correlations in terms of levels of gene expression between intermediates of signalling pathways and humoral defense factors, such as big defensin and lysozyme.

**Conclusion:**

Results of this study allowed the detection of modifications caused by QPX at the transcriptional level providing insight into clam immune response to the infection. These investigations permitted the identification of candidate genes and pathways for further analyses of biological bases of clam resistance to QPX allowing for a better understanding of bivalve immunity in general.

## Background

The hard clam, *Mercenaria mercenaria*, is exploited along the eastern coast of North America, from Maritime Canada to the Gulf of Mexico. This species is among the most commercially important bivalves in the United States and is well suited for aquaculture as it is characterized by a relatively fast growth. The hard clam is a sturdy bivalve and the only infectious agent that causes severe mortality episodes among wild and cultured clams is the protistan parasite QPX (Quahog Parasite Unknown) [1]. QPX is a unicellular protist member of the family Thraustochytridae [2]. Despite the ubiquitous nature of this family in aquatic environment, thraustochytrids were poorly studied and only few pathogens were described in this group [3]. QPX reported in 1989 at Prince Edward Island was linked to almost 100% of the mortality among cultured clams [4]. It was subsequently identified in other locations further south: Massachusetts in 1995 [5], Virginia in 1996 [6], New Jersey [7] and New York [8] in 2002, but the parasite was never detected further south than Virginia. Recent lab-controlled experiments [9] and *in situ *investigations [7,10] demonstrated variability of susceptibility among hard clam populations, with clams from southern broodstocks being more susceptible to QPX disease than northern broodstocks, suggesting a genetic origin of clam resistance. Field investigations also showed variability in the resistance toward QPX among different local (New York State) clam broodstocks [9]. Differences of susceptibility to pathogen infection between different populations were previously observed in other bivalves [11,12]. Intra-specific genetic variation in disease susceptibility to *Perkinsus marinus *was indirectly demonstrated by the evolution of resistance in disease-challenged natural populations of oysters [13].

Like other invertebrates, bivalves lack adaptive immunity and instead rely on various innate defenses against invading pathogens. In hard clam, hemocytes constitute the primary line of defense against materials recognized as non-self [14]. The presence of non-self materials in tissues initiates a complex molecular signalling cascade to stimulate cell-mediated immune responses, mainly involving phagocytosis or encapsulation of foreign materials, and the production of reactive oxygen species (ROS) [15,16]. Humoral factors, such as defensins, also play an important role because they possess various anti-microbial properties [17,18]. Enzymes, such as peptidases and lysozyme, are particularly crucial because of their ability to hydrolyze protein components of invading microorganisms [19-21]. Since bivalves have an open circulatory system, antimicrobial constituents associated with plasma and hemocytes are virtually present in all tissues. Histological observation of naturally- and experimentally-infected clams by QPX demonstrated that some individuals are sometimes able to mount a defense reaction characterized by an intense inflammatory response, phagocytosis (rare) and encapsulation of parasite cells. The presence of dead and necrotic QPX cells was reported in some instances, suggesting that clam's humoral and/or cellular response was sufficiently efficient to lead to the healing of infected individuals [6,8,22]. Histological observations of infected clam tissues and *in vitro *cultures also revealed an abundant production of a mucofilamentous net by QPX. These secretions are suspected to represent virulence factors that protect the parasite from host defense mechanisms [5]. *In vitro *investigations demonstrated that the mucus layer protects QPX from humoral defense factors in clam plasma and therefore, could be important to the establishment, as well as the development, of the disease [23]. However, clam immune response to QPX cells and/or mucus has never been investigated.

In bivalves, prior studies focusing on the identification of immune-related genes were performed in oysters [24-28], mussels [29] and scallops [30-34]. Molecular bases of defense mechanisms in hard clams, especially during QPX infection, are unknown. The only investigations that have studied molecular aspects of clam immunity were performed in the genus *Tapes *or *Ruditapes*; [35-37], a relatively distant member of the family Veneridae. Identification of immune-related genes involved in the response of *M. mercenaria *to QPX infection could lead to the development of tools that will contribute to the selection of resistant populations of clams and develop knowledge about clam immunity by the generation of a nucleic database for the species.

This study aimed for a better characterization of clam's response to QPX infection by investigating differentially expressed genes following parasite challenge. Our study is the first to apply transcriptomic approaches in *M. mercenaria*. Suppression subtractive hybridization (SSH), as well as the construction of cDNA libraries of expressed genes associated with quantitative PCR, was used to identify genes related to the defense of hard clam against its parasite.

## Results

### Identification of regulated genes in SSH libraries

The search for homology using the BLASTX program revealed a total of 25 unique sequences in gill libraries and 29 unique sequences in mantle libraries for *M. mercenaria *after 14 days of exposure, 10 unique sequences in gill libraries and 74 unique sequences in mantle libraries for *M. mercenaria *after 48 days of exposure, including sequences corresponding to known genes or unidentified ESTs. Genes regulated by QPX challenge were assigned to 7 major cellular physiological functions using the Gene Ontology (GO) Database: 1) stress response and detoxification; 2) cell communication, immune system and membrane receptors; 3) cell cycle regulation, DNA repair, protein regulation and transcription; 4) cytoskeleton production and maintenance; 5) respiratory chain; 6) metabolism; 7) ribosomal proteins; 8) unknown functions and 9) unidentified sequences. Up-regulated and down-regulated sequences in gill and mantle tissues are listed in tables 1 and 2, respectively. Sequences were submitted to NCBI with the following accession numbers: [GenBank: GO915165 – GO915272].

**Table 1:** Upregulated genes identified in mantle and gill tissues 14 and 48 days after challenge with washed and unwashed QPX cells.

**Homolog proteins**	**Homolog species**	**BlastX value**	**Libraries**	**GenBank accession no**
**stress response and detoxification**		4% of sequences identified in libraries from mantle tissue		
		11% of sequences identified in libraries from gill tissue		
metallothionein	*Corbicula fluminea*	1e-11	Mantle (14 days)	GO915213
cytochrome P450 like TBP	*Nicotiana tabacum*	2e-07	Gills (14 days)	GO915227
71 kDa heat shock protein	*Haliotis tuberculata*	6e-53	Mantle (48 days)	GO915235
ferritin subunit	*Meretrix meretrix*	3e-50	Mantle (48 days)	GO915233
				
**cell communication, immune system and membrane receptors**		7% of sequences identified in libraries from mantle tissue		
receptor of Activated Kinase C 1	*Mya arenaria*	4e-44	Mantle (48 days)	GO915258
lysozyme (Chain)	*Enterobacteria phage T4*	3e-04	Mantle (48 days)	GO915259
big defensin	*Tachypleus tridentatus*	5e-10	Mantle (48 days)	GO915266
sialic acid binding lectin	*Helix pomatia*	1e-04	Mantle (48 days)	GO915267
C-type lectin A	*Chlamys farreri*	5e-05	Mantle (14 days)	GO915219
nicotinic acetylcholine receptor subunit type H	*Lymnaea stagnalis*	8e-14	Mantle (14 days)	GO915224
				
**cell cycle regulation, DNA repair, protein**		4% of sequences identified in libraries from mantle tissue		
**regulation and transcription**		26% of sequences identified in libraries from gill tissue		
CCAAT/enhancer binding protein	*Aplysia kurodai*	5e-19	Mantle (14 days)	GO915218
translation elongation factor 1-alpha	*Dreissena polymorpha*	3e-108	Mantle (14 and 48 days) and Gills (48 days)	GO915211
elongation factor 1 beta	*Plutella xylostella*	4e-21	Mantle (48 days)	GO915246
eukaryotic translation elongation factor 1 delta	*Bos taurus*	4e-12	Mantle (48 days)	GO915241
similar to H3 histone, family 3B	*Macaca mulatta*	9e-37	Mantle (48 days)	GO915263
				
**cytoskeleton production and maintenance**		4% of sequences identified in libraries from mantle tissue		
		1% of sequences identified in libraries from gill tissue		
Actin	*Chlamys farreri*	4e-67	Mantle (48 days)	GO915239
myosin (essential light chain)	*Macrocallista nimbosa*	3e-38	Mantle (48 days)	GO915247
Tropomyosin	*Balanus rostratus*	7e-15	Mantle (48 days)	GO915261
alpha tubulin	*Leishmania braziliensis*	2e-05	Mantle (48 days)	GO915252
beta-tubulin	*Halocynthia roretzi*	2e-59	Gills (14 days)	GO915225
transgelin 3	*Danio rerio*	1e-08	Mantle (48 days)	GO915232
				
**respiratory chain**		12% of sequences identified in libraries from mantle tissue		
		11% of sequences identified in libraries from gill tissue		
cytochrome oxidase subunit 1	*Ruditapes philippinarum*	2e-29	Mantle and Gills (14 and 48 days)	GO915255
cytochrome oxidase subunit 3	*Inocellia crassicornis*	3e-19	Mantle (14 and 48 days)	GO915234
NADH dehydrogenase subunit 4	*Ruditapes philippinarum*	3e-30	Mantle and Gills (48 days)	GO915245
ATP synthase subunit 6	*Ruditapes philippinarum*	3e-25	Mantle and Gills (14 days)	GO915223
				
**Metabolism**		6% of sequences identified in libraries from gill tissue		
zinc-dependent alcohol dehydrogenase	*Lysiphlebus testaceipes*	1e-33	Gills (14 days)	GO915228
				
**ribosomal proteins**		3% of sequences identified in libraries from mantle tissue		
		1% of sequences identified in libraries from gill tissue		
ribosomal protein L17A	*Argopecten irradians*	8e-11	Mantle (48 days)	GO915262
ribosomal protein L11	*Ictalurus punctatus*	2e-08	Mantle (48 days)	GO915248
ribosomal protein S2e	*Onchocerca volvulus*	8e-25	Gills (48 days)	GO915270
ribosomal protein S3a	*Crassostrea gigas*	5e-99	Mantle (14 days)	GO915220
				
**unknown functions**		1% of sequences identified in libraries from mantle tissue		
		29% of sequences identified in libraries from gill tissue		
putative senescence-associated protein	*Pisum sativum*	3e-34	Gills (14 days)	GO915231
hypothetical protein TTHERM_02141640	*Tetrahymena thermophila SB210*	2e-36	Gills (14 days)	GO915230
hypothetical protein TTHERM_00648850	*Tetrahymena thermophila SB210*	8e-09	Mantle (48 days)	GO915260
SJCHGC09076 protein	*Schistosoma japonicum*	4e-03	Mantle (48 days)	GO915249
				
**unknown genes**		16% of sequences identified in libraries from mantle tissue		
		11% of sequences identified in libraries from gill tissue		
7 sequences			Mantle (14 days)	GO915212, GO915214-17, GO915221-2
15 sequences			Mantle (48 days)	GO915236-38, 40, 42-44, 50-51, 53-54, 56-57, 64-65
2 sequences			Gills (14 days)	GO915226, GO915229
4 sequences			Gills (48 days)	GO915268-69, GO915271-72

**Table 2:** Downregulated genes identified in mantle and gill tissues 14 and 48 days after challenge with washed and unwashed QPX cells.

**Homolog proteins**	**Homolog species**	**BlastX value**	**Libraries**	**GenBank accession no**
**stress response and detoxification**		8% of sequences identified in libraries from mantle tissue		
HSP70	*Mytilus galloprovincialis*	1e-52	Mantle (14 days)	GO915169
71 kDa heat shock protein	*Haliotis tuberculata*	6e-53	Mantle (14 days)	GO915166
				
**cell communication, immune system and membrane receptors**		5% of sequences identified in libraries from mantle tissue		
hemocyte defensin	*Crassostrea gigas*	1e-05	Mantle (48 days)	GO915199
peroxisome proliferator-activated receptor	*Oncorhynchus keta*	7e-07	Mantle (14 and 48 days)	GO915177
thioester-containing protein	*Euphaedusa tau*	2e-08	Mantle (48 days)	GO915190
				
**cell cycle regulation, DNA repair, protein regulation and transcription**		3% of sequences identified in libraries from mantle tissue		
transcription factor AP-1	*Strongylocentrotus purpuratus*	2e-16	Mantle (48 days)	GO915178
translation elongation factor 1-alpha	*Dreissena polymorpha*	3e-108	Mantle (14 and 48 days)	GO915167
				
**cytoskeleton production and maintenance**		2% of sequences identified in libraries from mantle tissue		
Actin	*Cyrenoida floridana*	1e-82	Mantle (48 days)	GO915201
alpha tubulin	*Theromyzon tessulatum*	5e-40	Mantle (48 days)	GO915209
alpha tubulin a1	*Mesenchytraeus solifugus*	1e-09	Mantle (48 days)	GO915208
				
**respiratory chain**		1% of sequences identified in libraries from mantle tissue		
		1% of sequences identified in libraries from gill tissue		
cytochrome b	*Ruditapes philippinarum*	8e-91	Gills (14 days)	GO915174
cytochrome c subunit I	*Ruditapes philippinarum*	6e-25	Mantle (14 days)	GO915173
				
**metabolism**		1% of sequences identified in libraries from mantle tissue		
ADP/ATP carrier	*Leishmania mexicana amazonensis*	2e-05	Mantle (14 days)	GO915170
				
**ribosomal proteins**		4% of sequences identified in libraries from mantle tissue		
ribosomal protein L7	*Argopecten irradians*	8e-03	Mantle (48 days)	GO915202
ribosomal protein L19	*Crassostrea gigas*	9e-21	Mantle (48 days)	GO915205
ribosomal protein L24	*Danio rerio*	3e-12	Mantle (14 days)	GO915172
				
**unknown functions**		8% of sequences identified in libraries from mantle tissue		
SJCHGC02792 protein	*Schistosoma japonicum*	3e-12	Mantle (14 days)	GO915171
hypothetical protein DDBDRAFT_0167791	*Dictyostelium discoideum AX4*	1e-04	Mantle (14 days)	GO915168
hypothetical protein	*Monodelphis domestica*	2e-27	Mantle (48 days)	GO915196
similar to product in *Drosophila melanogaster*	*Schistosoma japonicum*	7e-04	Mantle (48 days)	GO915187
				
**unknown genes**		17% of sequences identified in libraries from mantle tissue		
		3% of sequences identified in libraries from gill tissue		
1 sequence			Mantle (14 days)	GO915165
23 sequences			Mantle (48 days)	GO915179-86, 88-89, 91-95, 97-98, GO915200, 03-04, 06-07, 10
2 sequences			Gills (14 days)	GO915175-76

### Identification of genes from hemocyte cDNA libraries

**Figure 1:**
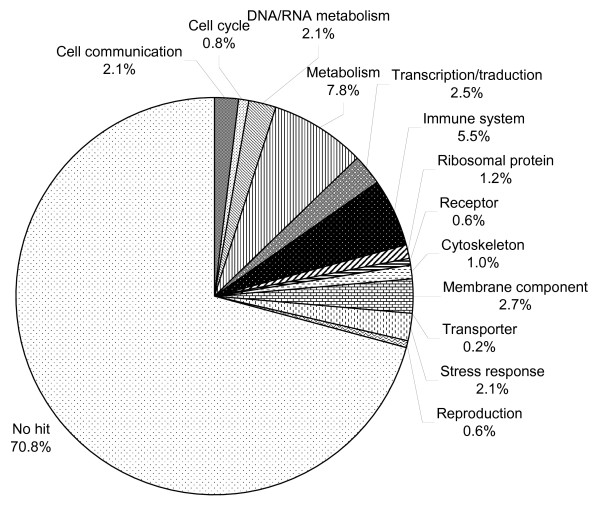
**Functional classification of the sequences identified in the hemocyte library (487 ESTs)**. Genes were clustered into 13 categories according to their putative biological function.

The sequencing of 1352 clones from the hemocyte cDNA library resulted in the characterization of a total of 487 ESTs that have been clusterized according to their function using the GO Database (Figure 1). Only 29% of these ESTs present an annotation and 71% remain unidentified. Several sequences presenting homologies with stress- and defense-related genes have been detected including Stress-Induced Protein STI [GenBank: GR209325] (BlastX value = 8e^-21^, *Cryptosporidium parvum *– [GenBank: XP_001388209]), Toll-Like Receptor TLR [GenBank: GR209327] (BlastX value = 2e^-4^, *Strongylocentrotus purpuratus *– [GenBank: XP_001201188]), Tumor Necrosis Factor Receptor-Associated Factor TRAF-6 [GenBank: GR209326] (BlastX value = 4e^-5^, *Chlamys farreri *– [GenBank: ABC73694]) and C1q – TNF related protein [GenBank: GR209324] (BlastX value = 3e^-2 ^*Danio rerio *– [GenBank: NP_001017702]).

### Expression patterns of differentially-regulated genes

**Figure 2:**
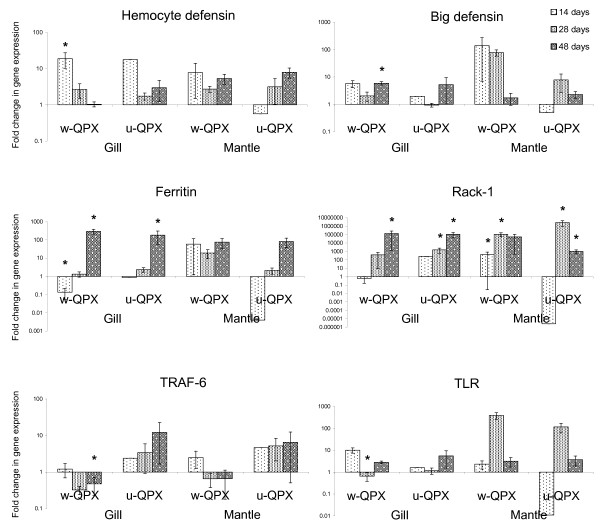
**Relative expression by quantitative PCR of selected transcripts from SSH (hemocyte and big defensins, ferritin, RACK-1) and hemocyte (TRAF-6 and TLR) libraries**. Expression levels were normalized to 18S RNA and presented as relative expression to controls (mean ± SD, n = 8 clams). * indicates significant differences of gene expression compared to controls at *p *< 0.05 (Student's *t*-test).

Results showed that QPX challenge induced significant changes in the expression of several of the eighteen investigated genes. This modulation was characterized by a highly variable regulation of these genes 14 days after challenge (Figure 2). At that sampling time, few transcripts were significantly regulated, particularly in w-QPX challenged clams, including hemocyte defensin, C1q, actin, HSP 70 and ferritin (Table 3). After 28 and 48 days, significant variations of gene expression were observed in both washed and unwashed-QPX challenged clams with a high variability according to the challenge and the tissue (Table 3, Figure 2). Some genes, such as TRAF-6, were more specific to the challenge (washed or unwashed-QPX), whereas other genes tended to be more linked to particular tissues such as the big defensin (Figure 2). In addition, some gene expressions presented continuous trends over time (decrease or increase) as TRAF-6 and ferritin, while other genes displayed strong modulation after 28 days (TLR expression in mantle tissues, Figure 2). Multifactor analysis indicated significant effects of all individual parameters (time, treatment and tissues) on actin and big defensin expression and a significant effect of combined parameters on AP-1, lysozyme and TLR expression (Table 4).

**Table 3:** Summary of the results of Student's t-tests of gene expression data.

	14 days				28 days				48 days			
			
	Mantle		Gills		Mantle		Gills		Mantle		Gills	
						
	w-QPX	u-QPX	w-QPX	u-QPX	w-QPX	u-QPX	w-QPX	u-QPX	w-QPX	u-QPX	w-QPX	u-QPX
hemocyte defensin			**+**									
big defensin											**+++**	
lysozyme					**+**		**++**				**+**	**---**
C1q – TNF related protein			**+**									
TRAF-6											**--**	
Toll-like receptor							**-**					
RACK-1	**+++**				**+++**	**+++**		**+**		**+**	**++**	**+++**
peroxisome proliferator-activated receptor					**+**							**+**
HSP70			**-**			**+**						
stress-induced protein STI					**+**							
ferritin			**-**								**++**	**+**
metallothionein						**++**					**-**	**+++**
actin			**+**		**++**							
AP-1					**++**		**+**				**++**	
elongation factor beta	**+++**								**-**		**---**	**+++**
NADH sub-unit IV								**+**				
senescente associated protein											**--**	**+**
cytochrome P450 like TBP	**+++**								**-**			

Symbols + and - respectively indicate significant increase or decrease of gene expression compared to controls and the number of symbols for each condition refers to the *p*-value: + or -: *p *< 0.05, ++ or --: *p *< 0.01 and +++ or ---: *p *< 0.001.

**Table 4:** Effects of QPX challenge, sampling time and tissue type on gene expression in *M. mercenaria *(Multifactor ANOVA followed by Holm-Sidak post-hoc test).

	Time	condition	tissue	time × condition	time × tissue	condition × tissue	time × condition × tissue
hemocyte defensin					*		
big defensin	***	*	**	**	***		**
lysozyme				*			*
Toll-like receptor	***		*	*	***		*
ferritin	***						
actin	**	*	**	***	**	*	***
AP-1	*			*	*		**
NADH sub-unit IV	*				*		
senescente associated protein	***		*	*	***		

Only genes showing significant variations are presented. Symbols refer to the *p*-value: *: *p *< 0.05, **: *p *< 0.01 and ***: *p *< 0.001.

**Figure 3:**
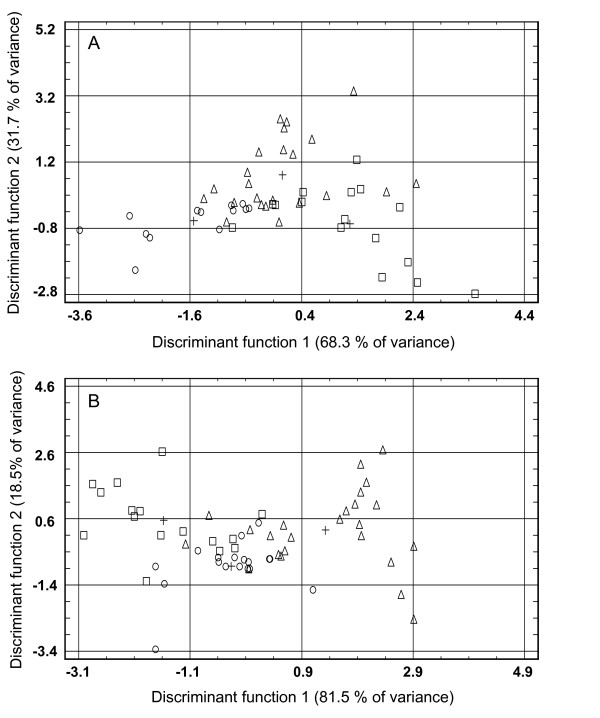
**Scatter plots of Discriminant Analysis scores in mantle (A) and gill (B) tissues for un-challenged controls (circles) and clams challenged with washed (triangles) or unwashed (squares) QPX cells**. Positions of group centroids for each treatment are indicated by a black cross.

Discriminant Analysis performed on data from mantle and gill tissues (samples from different time points combined) revealed significant discrimination of the treatments in both tissues by Function 1 (*p *= 0.05 and 0.01, respectively) which explained 68.3% and 81.5% of the total variance respectively (Table 5). Scatter plots of discriminant functions indicated a small overlapping between treatments with a good discrimination of centroids by Function 1 (Figure 3). Examination of the structure correlation matrix (Table 6) revealed that 9 variables were highly loaded in Function 1 for both mantle and gill tissue analyses. Among these variables, the expression of TLR, AP-1, big defensin and lysozyme were highly correlated (Table 7, *p *< 0.001).

**Table 5:** Gene expression data from different sampling times were combined.

Tissue	Discriminant	Eigenvalue	relative	Canonical	Wilks	Chi-	Degrees of	Statistical
	function		percentage	Correlation	Lambda	Square	freedom	significance
Mantle	1	1.18	68.3	0.74	0.30	50.4	36	0.05
	2	0.55	31.7	0.59	0.65	18.1	17	0.38
Gills	1	1.54	81.5	0.78	0.29	57.2	36	0.01
	2	0.35	18.5	0.51	0.74	13.9	17	0.67

**Table 6:** Structure matrix of Discriminant Analyses on gene expression data obtained from mantle and gill tissues.

	mantle tissue		gill tissue	
		
	function	function	function	function
	1	2	1	2
Toll-like receptor	1.118*	1.021	2.109*	-1.269
AP-1	4.635*	0.899	1.492*	-0.098
peroxisome proliferator-activated receptor	-0.356*	0.119	-2.771*	1.871
big defensin	-1.164*	-0.197	2.178*	-0.883
lysozyme	-2.662*	0.220	-0.704*	0.163
metallothionein	6.612*	-2.521	0.313*	0.241
Actin	-2.452*	-1.006	-2.161*	1.222
NADH sub-unit IV	0.335*	0.073	-1.133*	1.051
senescence associated protein	-0.095*	0.036	0.352*	0.025
hemocyte defensin	1.642*	0.238	0.461	0.775
HSP70	1.413*	-0.295	0.377	-0.613
elongation factor beta	-6.159*	-0.112	-2.472	2.639
cytochrome P450 like TBP	-0.155	-0.270	-0.107*	-0.009
TRAF 6	0.322	-0.364	-1.859*	-0.946
stress-induced protein STI	-1.698	2.122	5.207*	-3.063
RACK-1	0.1242	-0.645	0.033	0.181
C1q – TNF related protein	0.278	0.857	0.142	-0.559
Ferritin	0.115	0.139	-0.430	0.992

Largest absolute correlations between variables and discriminant functions are indicated by *.

**Table 7:** Pearson's correlation coefficients of genes related to cell signalling (AP-1, TLR, TRAF-6; RACK-1) and humoral defense factors (hemocyte and big defensins, lysozyme).

	lysozyme	big defensin	hemocyte defensin
			
AP-1	0.934	0.736	-0.025
	(5.3e^-51^)	(-2.5e^-20^)	(NS)
Toll-like receptor	0.408	0.726	-0.030
	(7.9e^-6^)	(1.4e^-19^)	(NS)
TRAF 6	-0.034	0.016	0.087
	(NS)	(NS)	(NS)
RACK-1	-0.015	-0.039	-0.020
	(NS)	(NS)	(NS)

*P*-values are indicated between parentheses and non significant correlations are indicated by NS.

## Discussion

Our investigations allowed the identification of components involved in different physiological processes related to signal transduction (RACK-1, TLR, TRAF-6), stress response (HSP, metallothionein, ferritin), immunity (lectins, defensins, lysozyme) and protein synthesis (transcription and elongation factors). This new information allowed the detection of modifications caused by QPX at the transcriptional level. Our results depict some integrative aspects of clam responses to QPX infection and more specifically to different forms of QPX challenges (washed versus unwashed cells of the parasite).

Since QPX is an opportunistic parasite usually found in pallial tissues (gills and mantle) of infected clams [7], we focused our investigations on these tissues to identify differentially expressed genes in response to the infection. Interestingly, the comparison of gene distribution from subtracted libraries indicated some variations according to the tissue and the time of sampling. After 14 days of QPX challenge, similar numbers of unique genes were identified in mantle and gill tissues. However, 48 days post-challenge, a clear difference was observed among tissues with a larger proportion of genes identified in mantle as compared to gills. In addition, 24% of identified genes in mantle tissues presented homologies with proteins known to be involved in stress and immune response, whereas only 11% of identified genes in gills were involved in stress response (Tables 1 and 2). These results suggest that molecular changes observed 14 days after challenge represented a systemic acute response of clams resulting from the injection of parasite in the circulation, whereas the differences observed after 48 days could be related to clam response to established QPX infections in mantle tissues. These results fit well with histological observations made following injection of QPX into the pericardial cavity of hard clams that showed an early (2 weeks) systemic distribution of QPX in clam tissues, followed by the development of most intense lesions in mantle tissues [22]. It is noteworthy that the development of QPX lesions following intrapericardial injection matches well, temporally (>4 weeks) and spatially (organs infected), with the typical disease development in naturally infected clams [22].

Our investigations demonstrated the modulation in QPX-challenged clams of components with strong homologies to stress-related proteins, including heat shock proteins (HSP's) and their co-chaperones STI1 (Stress Induced Protein), metallothionein and ferritin. These molecular chaperones protect the cell and maintain homeostasis under stressful conditions [38]. HSPs were identified in several bivalve species in response to various chemical, physical and pathogenic stresses and appear to represent a general marker of non-specific stress [25,39-41]. Previous studies demonstrated that heat-shock proteins were able to stimulate innate immune system in mammalians [42]. Our results are consistent with previous studies, which showed that the relationship between HSP and defense response was not established, despite the identification of heat-shock proteins following pathogenic challenge in invertebrates [25,36,43]. HSP 70 was significantly repressed in gill tissues of w-QPX challenged clams after 14 days and up-regulated in mantle tissues of u-QPX challenged clams (unwashed cells) after 28 days (Table 3). However, Multifactor ANOVA analysis indicated no significant role of HSP expression in the response of clams to the different treatments (Table 4). MT's are an ubiquitous class of metal-binding proteins that function in the homeostasis of essential metals, as well as serving a detoxification role by sequestering toxic metals. In oysters, Anderson et al. [44] demonstrated that MT's were able to scavenge reactive oxygen species. In mammals, immune stimulants have been shown to be effective inducers of MT's [45]. However, the role of MT's in invertebrates, and especially in bivalves, appears more complex. Bacterial challenge induced a repression of MT expression in *Crassostrea virginica *[26] but not in the bay scallop *Agropecten irradians *[46] or during trematode parasitism in the cockle *Cerastoderma edule *where MT concentration increased [47]. Differences of MT expression could be related to the nature of the pathogen and its capacity to produce toxic compounds against its host. In our study, MT was significantly up-regulated in mantle after 28 days and gill tissues after 48 days in clams challenged by unwashed QPX but not in w-QPX challenged clams (Table 3). Discriminant analysis in mantle and gill tissues also revealed the specific importance of MT to separate the treatments in Function 1 (Table 6). Ferritin has also been classified as a stress protein due to its similarity with proteins involved in detoxification processes [24]. However, ferritin was also associated with defense mechanisms because of its role in the regulation of iron availability to infectious agents [48]. Previous studies demonstrated an increase of ferritin expression in invertebrates following exposures to pathogen-associated molecular patterns (PAMPs) or bacterial challenge [24,49,50]. In our study, quantitative PCR revealed an increase of ferritin expression in clams challenged by QPX after 28 and 48 days (Figure 2). However, significant increase of ferritin expression was only observed in gill tissues (Table 3). The biological significance of changes in ferritin expression levels is not known in clams since the effects of iron on QPX has never been investigated, but Gauthier and Vasta [51] demonstrated limited *in vitro *growth of the oyster pathogen *Perkinsus marinus *under low iron concentrations.

Subtracted libraries also allowed the identification of genes coding for several proteins involved in humoral defense including lysozyme, lectins and defensins. Lysozyme is a well-known protein possessing anti-microbial activities; lysozyme activity has been detected in the body fluids and tissues of many bivalve mollusks and is believed to play a role in host defense and digestion [19,52,53]. Different results have been previously reported on the variation of lysozyme concentration in response to parasites in mollusks. Lysozyme concentrations were unchanged in clams *Tapes decussatus *infected by *Perkinsus atlanticus *[54] and in oysters *Crassostrea virginica *infected by *P. marinus *[55,56]. However, a subsequent investigation in oysters showed a slight decrease in lysozyme concentration in *P. marinus*-infected oysters [57]. Our results indicate a differential response according to the treatment since the injection of washed QPX cells (w-QPX) induced significant expression of lysozyme in mantle and gill tissues, whereas unwashed parasite cells (u-QPX) caused a down-regulation of lysozyme expression in gill tissues after 48 days (Table 3).

Two different lectins were also identified in up-regulated libraries (Table 1). Lectins play an important role in invertebrate immunity as non-self pattern recognition molecules by promoting agglutination and opsonization of pathogens. In Manila clams *Ruditapes philippinarum*, Kang et al. [35] demonstrated significant increase in lectin expression following pathogen challenge. Interestingly, lectins isolated from *R. philippinarum *[58] and oysters *C. virginica *[59] bind to the surfaces of purified hypnospores from *Perkinsus sp*. enhancing their phagocytosis by hemocytes. Identified lectins in libraries could as well be associated to the activation of the complement pathway since a thioester-containing protein (TEP) was also found in subtracted libraries (Table 2). Complement pathway is activated in reaction to the presence of PAMPs, leading to increased opsonization and phagocytosis activity by defense cells [60]. Several TEP with homologies with _α_-macroglobulin were previously characterized in other invertebrates [61,62] and in bivalves [63,64]. A transcript with homologies with C1q was also present in our libraries. C1q is the target recognition of the classical complement pathway that is crucial for the clearance of pathogens in vertebrates [65] and invertebrates [66].

Our approach also led to the identification of two different defensins that show different expression patterns in mantle and gill tissues. Defensins are small antimicrobial peptides (AMP) and represent major actors in innate immunity [67]. Defensins were isolated from mussels [68], scallops [33] and oysters [69,70]. These AMPs can be constitutively expressed, as observed in oysters [70], or induced in response to infection, as in scallop [33]. Most previously described defensins were characterized from hemocytes but some were also constitutively expressed in pallial tissues [69]. In our study, quantitative PCR revealed that the hemocyte defensin was significantly up-regulated in gills only after 14 days, while no significant change was observed in mantle, despite the tendency to an increase of the hemocyte defensin expression after 48 days following challenge with washed and unwashed QPX cells (Figure 2). Initial induction of the hemocyte defensin after 14 days in gills could reflect systemic hemocyte activity as gill tissues are normally rich in hemocytes compared to mantle. Later on, increased PCR signals in mantle tissues could be related to increased expression in hemocytes present near infection sites or might simply reflect the mobilization of hemocytes toward active infection sites as part of the normal inflammatory response [5,8]. This may, in turn, lead to the observed shift of gene expression among tissues. Similar patterns were found in other studies investigating defensin expression in oysters [70]. Regarding the big defensin, a significant induction was observed after 48 days in gill tissues of clams challenged with washed QPX whereas a tendency to a decrease of defensin expression was noticeable in mantle tissue (Figure 2). Moreover, the big defensin appeared as an important variable to discriminate treatments in gill and mantle tissues (Table 6). Defensins present a great diversity in terms of structural features, biological properties and functions, and also in their tissue distribution and expression. Defensins from *C. gigas *exhibit high activities against gram positive bacteria but low activity against fungi [69], whereas big defensins from *A. irradians *and the horseshoe crab *Tachypleus tridentatus *exhibit strong fungicidal activities [33,71]. Trends of both defensins suggest a certain level of specificity in the response of *M. mercenaria *to washed and unwashed QPX cells in mantle and gill tissues.

Several genes corresponding to membrane receptors and elements of pathways involved in defense responses have also been identified in our libraries. Among them, RACK-1 is involved in the protein kinase C (PKC) pathways and acts as an activator/receptor for this protein [72]. RACK-1 plays a key role as the crossroad among several cellular pathways in cell communication by acting as a scaffold protein on the translocation of the signalling proteins towards the membrane-bound receptors [72]. Ron et al. [73] demonstrated *in situ *association of RACK-1 and PKC during phorbol 12-myristate 13-acetate (PMA) challenge, an activator of reactive oxygen species production [74,75]. Overexpression of RACK-1 also led to enhanced spreading and increased focal adhesion in mammalian cells [76]. These results suggested an involvement of RACK-1 in phagocytosis and ROS production. RACK-1 was previously identified in bivalves exposed to pollutants, physical stress and pathogens [36,41,77]. In the hard clam, our quantitative PCR results revealed that QPX challenge significantly induced the expression of RACK-1 in both gill and mantle tissues (Figure 2 and Table 3). Libraries generated from hemocytes also led to the identification of several elements of the NF-kB pathway. Toll-like receptors (TLRs) are among the most important families of pattern recognition receptors (PRRs) and have already been identified in other bivalves [25,30]. They are able to selectively recognize and initiate the response against a large number of varied and complex PAMPs [78]. Tumor necrosis factor receptor-associated factor (TRAF), another component of the NF-kB pathway, was also detected in our hemocyte libraries. This intermediary possesses a unique receptor-binding specificity that results in its crucial role as the signalling mediator for both the TNF receptor superfamily and the TLR superfamily [79]. Activation of this pathway induces expression of immune response genes triggered by transcriptional activator proteins. Among them, the transcriptional factor AP-1 was identified in our subtracted libraries. Interestingly, our results demonstrated an important involvement of NF-kB components in the differential response to washed and unwashed parasite cells (Tables 4 and 6), as well as a high correlation (*p *< 0.001) between humoral defenses (lysozyme, big defensins), TLR and the transcriptional factor AP-1 (Table 7). These results suggest that activation of the NF-kB pathway occurred following the recognition of QPX by TLR and the activation of AP-1, leading to a specific response characterized by the production of humoral defense factors including lysozyme and the big defensin.

Actin is often used as a house-keeping gene but we clearly observed a modulation of this gene in gills (14 days) and mantle (28 days) tissues following challenge with washed parasite cells (Tables 3, 4 and 6). Actins are highly conserved proteins that are ubiquitously expressed in all eukaryotic cells. They are involved in the formation of filaments that are major components of the cytoskeleton and participate in many important cellular functions including cell motility, organelle movements and cell signalling [80,81]. With regard to infections, actin was, up-regulated in *Biomphalaria tenagophila *at a proteomic level in the presence of *Schistosoma mansoni *[82]. The involvement of actin in QPX disease pathogenesis, if any, is unclear but it may participate in the encapsulation of parasite cells by host hemocytes leading to healing as in other host-parasite models [83].

Results of quantitative PCR also indicated that some genes were differentially regulated according to analyzed tissue (TLR, big defensin, Table 4) or inoculum (big defensin, Tables 4 and 6). Discriminant Analysis revealed the importance of signalling pathways and humoral defenses to differentiate between QPX-challenged and unchallenged clams or between clams injected with washed or unwashed parasite cells (Figure 3 and Table 6). It should be mentioned that, because of sample size requirements of the statistical test, Discriminant Analyses were performed on data obtained throughout the experiment by pooling samples collected at 14, 28 and 48 days post-challenge. Such a holistic approach eliminated the effect of genes that displayed high temporary modulations within each treatment and might neglect certain specific clam responses. This limitation could explain the barely significant results obtained with Discriminant Analyses in mantle tissues (Table 5). Despite this drawback, our analyses discriminated between clams injected with washed or unwashed parasite cells, highlighting the importance of QPX mucus during host-pathogen interactions. QPX mucus was suggested to represent a virulence factor that protects the parasite from host's cellular and humoral defense mechanisms [5]. Proteases were also detected in QPX mucus [84] and Thrautochytrids are known to produce several proteolytic enzymes as extracellular products [85]. Injection of unwashed QPX could protect the parasite from constitutive defenses of hard clams and enhance their ability to establish infection within host tissues, as well as prevent the detection of the parasite's PAMP's, thereby limiting the response of hard clams. In contrast, washed cells can be more readily phagocyted or encapsulated by hemocytes and neutralized by humoral factors [23]; they can also present PAMPs on their surface, enhancing clam's immune response. Thus, injection of washed QPX cells could induce an efficient defense response in clams, leading to elimination of parasites and failure of disease establishment.

## Conclusion

In conclusion, this study is the first to characterize molecular modulation in clams in response to QPX infection. A large number of new candidate genes was identified including several genes involved in stress and defense response and cell signalling. Quantitative PCR revealed significant changes in the expression of some of these genes in response to QPX challenge, as well as some correlation between gene expression of intermediates of signalling pathway and humoral defenses. Additional experiments are needed to further characterize molecular components involved in *M. mercenaria *response to its parasite. Specifically, further experiments should compare gene expression in susceptible and resistant clam broodstocks. Generated sequence information could also contribute to the construction of the first hard clam micro-array necessary for investigating gene expression on a larger scale.

## Methods

### QPX cultures

QPX strain NY0313808BC7 was isolated from nodules of infected New York clams [86] and subcultured in a culture medium based on muscle homogenates from hard clams adjusted at 1000 μg.mL^-1 ^of proteins in filter-sterilized artificial seawater (FASW) [87]. QPX cultures were initiated in 25-cm^2 ^flasks incubated at 23°C for 1 week to reach the exponential phase of growth. Parasite cultures were thereafter subdivided into two aliquots: one aliquot was untreated resulting in QPX cells associated with their typical abundant mucus secretions surrounding parasite cells (cells and mucus – u-QPX) and another aliquot was washed according to a protocol adapted from Anderson et al. [23] to remove the mucus from cells (w-QPX). Briefly, a volume of QPX culture was mixed well by repeatedly drawing up and expelling the culture with a 3 mL-syringe without a needle. A small volume of well-mixed culture was then suspended in five times its volume of sterile culture medium. This suspension was then vortexed for 10–15 seconds and centrifuged for 15 min at 600 × g [23]. The supernatant was removed and the QPX pellet was then washed two times and resuspended in sterile culture medium. This washing procedure has been thoroughly tested and found not to affect QPX viability [88]. QPX biomass in each aliquot was then measured using a semi-automated fluorometric technique according to Buggé and Allam [88] and QPX suspensions were adjusted with sterile culture medium to obtain the same parasite biomass.

### Hard clams and experimental infections

QPX-free adult *Mercenaria mercenaria *were obtained from Frank M. Flowers Oyster Company (Oyster Bay, NY). Clams were acclimated one week in the laboratory, held in 150-L tanks with re-circulating water (28–30 ppt) at 21 ± 1°C and fed daily with commercial algae (DT's Live Phytoplankton, Sycamore, IL). After acclimation, clams were divided into three groups of 30 individuals and challenged with either washed (w-QPX) or unwashed (u-QPX) parasite cells to compare clam response in presence or absence of the mucus layer surrounding QPX cells. Experimental infections were performed according to Dahl and Allam [22] by injecting 100 μL of culture medium containing 5 × 10^4 ^QPX cells into clam's pericardial cavity. Control clams were injected with 100 μL of sterile culture medium. Following injection, clams were maintained out of the water for 1.5 h and were thereafter transferred to separate tanks. Mortality was monitored daily. For each experimental condition, 8 clams were sampled at 14, 28 and 48 days after challenge. Hemolymph was withdrawn from the adductor muscle and held individually on ice. Samples were centrifuged at 700 × g for 10 min at 4°C, plasma was discarded and hemocyte pellets were rapidly frozen in liquid nitrogen before storage at -80°C. Concomitantly, gill and mantle tissues were dissected and frozen individually until RNA extraction.

### RNA extraction

Total RNA was extracted from hemocyte pellets and clam tissues using TRI^® ^Reagent (Invitrogen, Carlsbad, CA, USA). Polyadenylated RNA was isolated using the PolyATtract^®^mRNA Isolation System (Promega, Madison, WI, USA) according to manufacturer's instructions. Messenger RNAs were resuspended in RNase-free water and both quantity and quality were assessed by spectrophotometry (OD260, OD280).

### Suppression subtractive hybridization

**Figure 4:**
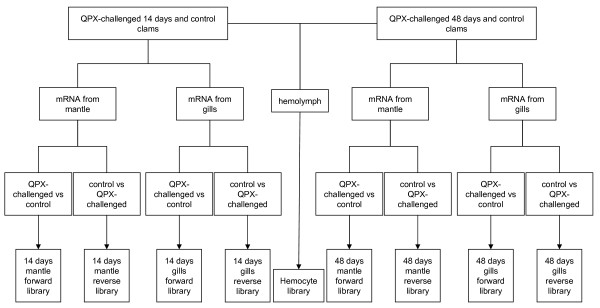
**Diagram of the different subtractions and cDNA libraries performed in *M. mercenaria***. Clams challenged with washed and unwashed QPX cells were pooled to perform SSH. The hemocyte library was generated from a pool of all challenged and unchallenged clams collected at 14 and 48 days.

The suppression subtractive hybridization technique (SSH) [89] was used to identify genes involved in clam's immune response following QPX challenge. Messenger RNAs isolated from gill and mantle tissues at 14 and 48 days were pooled for each treatment and sampling time. Both forward and reverse subtracted libraries were generated on 2 μg of pooled mRNA for each SSH library construction (Figure 4). First and second strand cDNA synthesis, *Rsa*I endonuclease enzyme digestion, adapter ligation, hybridization, and PCR amplification were performed as described in the PCR-select cDNA subtraction manual (Clontech, Palo Alto, CA, USA). Differentially expressed PCR products were purified and cloned into pGEM-T vector (Promega, Madison, WI, USA). Bacteria (DH5α phage resistant) were transformed and cultured in Luria-Bertani medium (with 100 μg.L^-1 ^ampicillin, final concentration). Vectors from two hundred colonies per library were extracted using an alkaline lysis plasmid minipreparation, and screened by size after digestion. A total of 298 clones from forward and reverse libraries were sequenced using an AB3100 sequencer (Perkins-Elmer) and Big Dye Terminator V3.1 Kit (Perkins-Elmer).

### Full-length cDNA library construction

Messenger RNAs isolated from hemocytes were pooled and cDNA library was generated using Creator™ SMART™ cDNA Library Construction Kit (Clontech, Palo Alto, CA, USA) according to manufacturer's instructions. The cDNA library was cloned into the pDNR-LIB Vector and transformed in bacteria (DH5α phage resistant) and cultured in Luria-Bertani medium (with 100 μg.L^-1 ^ampicillin, final concentration). A total of 1352 clones were randomly selected and sequenced using an AB3100 sequencer (Perkins-Elmer) and Big Dye Terminator V3.1 Kit (Perkins-Elmer).

### Sequence analysis

The ABI sequence data were basecalled using 'phred'  and subsequently clipped for bad-quality and vector using 'lucy' (compbio.dfci.harvard.edu/tgi/software) with standard parameters. cDNA sequences were automatically screened against UniProt (BLASTX) and all ESTs from EMBL (BLASTN).

### Real-Time PCR Analyses

Fourteen genes were selected from SSH libraries for further investigations of their expression including 3 stress-related genes (metallothionein, HSP 70 and ferritin), 3 defense-related genes (big defensin, hemocyte defensin and lysozyme) and 2 genes involved in cell signalling (receptor of activated kinase C1, peroxisome proliferator-activated receptor). In addition, several transcripts involved in gene regulation, transcription factors, the cytoskeleton and metabolism were analyzed (elongation factor 1 beta, transcription factor AP-1, actin, NADH4). Two transcripts (senescence-associated protein, cytochrome P450 like TBP) previously identified in other bivalves during parasite challenges [25] and in our libraries were also selected. Four additional transcripts encoding for stress related gene (stress-induced protein – STI1) and components of the NF-kB pathway (tumor necrosis factor receptor-associated factor – TRAF-6, Toll like receptor – TLR) and complement system (C1q-TNF related protein) were selected from the hemocyte library. Expression of all candidates was compared to controls in gill and mantle tissues at 14, 28 and 48 days post-challenge. For each sample, 10 μg total RNA was individually submitted to reverse transcription using the oligo dT anchor primer (5'-GACCACGCGTATCGATGTCGACT_(16)_V-3') and Moloney murine leukaemia virus (M-MLV) reverse transcriptase (Promega, Madison, WI, USA). The real-time PCR assay was performed with 3 μL cDNA (1/20 dilution) in a total volume of 10 μL, using a Chromo 4™ System Q-PCR (Bio-Rad, Hercules, CA USA). Concentrations of the reaction components were as follows: 1× Absolute QPCR SYBR Green ROX Mix (ABgene, UK) and 70 nM of each primer. Primer sequences of the 18 genes selected in the *M. mercenaria *SSH and hemocyte libraries are presented in Table 8. Reactions were realized with activation of Thermo-Start^® ^DNA polymerase at 95°C for 15 min followed by amplification of the target cDNA (50 cycles of denaturation at 95°C for 30 sec, annealing and extension at 60°C for 1 min) and a melting curve program from 95 to 70°C that decreased the temperature by 0.5°C every 10 sec. Readings were taken at 60°C. PCR efficiency (E) was determined for each primer pair by determining the slope of standard curves obtained from serial dilution analysis of cDNA from different experimental samples (treatment and control). The comparative CT method (2-ΔΔCT method) was used to determine the expression level of analyzed genes [90]. The expression of the candidate genes was normalized using ribosomal RNA 18S fragment as a housekeeping gene by the specific primers (Table 8). Fold units were calculated on normalized expression values by dividing gene expression in tissues from challenged clams by controls. Results are given as the mean and standard deviation of eight replicates per condition.

**Table 8:** Combinations of primers used in quantitative PCR assays.

***Gene name***	***Gene function***	***Primer sequences***
18S	Ribosomal protein	F: CTGGTTAATTCCGATAACGAACGAGACTCTA
		R: TGCTCAATCTCGTGTGGCTAAACGCCACTTG
Hemocyte defensin	Immune system	F: ACAAATGTAACAGGCATTGTAGGAGCAT
		R: CATGTGCATCTTCGGTAAAAAGTCCA
Big defensin	Immune system	F: ATGGACACTAGGAAAGTCTACTGTGTGCT
		R: ACAAGTGCAACCCAGACCCAAGGTGA
Lysozyme	Immune system	F: ATAACGAAAGACCAAGCTCGTGCTCT
		R: GTTTTGGGTCCTAGATCTCCCCTGTA
C1q – TNF related protein	Immune system	F: ATGCAAGTCAGTGCCGTGATACACCCAGA
		R: AATAAAGCGCCACTGAAAGTTGTTCCATG
TRAF-6	Immune system	F: GAACTAGCAAACAGGAATTGGGAGGCGCT
		R:GTCAAGTGATGGCTCATCTTGGATGCTGC
Toll-like receptor	Immune system	F: GTAACAAATTTCACTCTGGCCGCTGACGC
		R: TAGCTGAAATCCAACGACTGCACCCGTAA
RACK-1	Cell communication	F: CCTAACAGATACTGGCTGTGTGCTGC
		R: GTCTGTCCATCTGCGGACCATGCAAG
Peroxisome proliferator-activated receptor	Cell communication	F: CATAGCCAATTCCATACCCCTGGCCA
		R: AGTTGGCATCGCCACTGTCGCTGCTC
HSP70	Stress response	F: AATGACAAAGGCCGTCTCAGCAAGGA
		R: TCTAACCAACTGATGACCTCGCTACA
Stress-induced protein STI	Stress response	F: GAAGCTGTTGAACAAGCCAAGAGTGGAGC
		R: GTCTCTTGAATTCGGGGATCTTGAGCTGC
Ferritin	Iron transport	F: ATGTCTGTTTCACGACCTCGACAGAA
		R: AGTTTCTCGGCATGCTCACGTTCCTC
Metallothionein	Detoxification	F: ACCAGTGATGGTGGCTGCAGGTGTGG
		R: TTACACGAACAGCCACTATCACACTG
Actin	Cytoskeleton	F: ATTGTGATGGACTCTGGTGATGGTGT
		R: TCTCTAACAATTTCTCTCTCAGCCGTTGT
Transcription factor AP-1	Transcription	F: AGAAAACTTGAAAGAATTGCGCGACT
		R: TGTGACATCATTATCTGGCACCCACT
Elongation factor beta	Transcription	F: CCTTGGGATGATGAAACAGATATGGC
		R: CTAATCTTGGCATCTTCTATAACAGC
NADH sub-unit IV	Mitochondrial respiration	F: CCGTGGGATTTAGGGAGGGATAATATGCT
		R: ACTCCAGTTAACAACATTGATCCCCTCAA
Senescence associated protein	unknown	F: AACCTGTCTCACGACGGTCTAAGCCCAGC
		R: TTACCACAGGGATAACTGGCTTGTGG
Cytochrome P450 like TBP	unknown	F: GTCTGGAAAACGGCCACAAGGCACCT
		R: TTATACAAGGTAACCGGCTTGGACGC

### Statistical analysis

Variations in gene expression levels in tissues from clams submitted to different treatments were analyzed using Student's *t*-test using SigmaStat Version 3.10 (Systat Software, Inc). Effects of sampling times, treatments and tissues on gene expression were analyzed using multifactor analysis of variance (ANOVA) followed by a Holm-Sidak post-hoc test when appropriate. Correlation analyses of the expression of different genes were made using Pearson's method. Finally, Discriminant Analysis (DA) of gene expression was performed using Statgraphics plus Version 2.1. As DA requires a minimum within-treatment sample size of 20, data from the different sampling times within each treatment were combined and DA was separately applied on mantle and gill tissue groups. DA determines linear combinations of variables (genes) that maximize differences among *a priori *defined groups (treatments). The relative contribution of each variable was assessed on the basis of the structure correlations to interpret the discriminating power of the independent variables. In all tests, differences were considered statistically significant at *p *< 0.05.

## Authors' contributions

MP carried out SSH and expression libraries, participated in the sequence alignment and drafted the manuscript. AT was in charge of molecular experiments, carried out real time PCR and helped in drafting the methodology section of the manuscript. BA is the lead PI on this work. He conceived the design of the study, coordinated experiments and supervised the statistical analysis of the results. All authors read and approved the final manuscript.
